# Tracking the British agricultural revolution through the isotopic analysis of dated parchment

**DOI:** 10.1038/s41598-022-26013-4

**Published:** 2023-01-09

**Authors:** Sean P. Doherty, Michelle M. Alexander, Stuart Henderson, Jason Newton, Jonathan Finch, Matthew J. Collins

**Affiliations:** 1 Department of Archaeology and History, University of Exeter, Exeter, UK; 2 Department of Archaeology, University of York, York, UK; 3 Department of Archaeology, University of Cambridge, Cambridge, UK; 4 NERC National Environmental Isotope Facility, Scottish Universities Environmental Research Centre, East Kilbride, UK; 5 Globe Institute, University of Copenhagen, Copenhagen, Denmark

**Keywords:** Palaeoecology, Stable isotope analysis, Environmental social sciences

## Abstract

Between the sixteenth and nineteenth century, British agriculture underwent a ‘revolutionary’ transformation. Yet despite over a century of research and the recognised centrality of agricultural developments to industrialisation and population growth, the character or chronology of any ‘revolution’ during this period remains contentious. Enquiry has been hampered by the fragmented and locally specific nature of historic accounts and the broad dating of early-modern zooarchaeological assemblages. To address this, we conducted stable isotope analysis on 658 legal documents written on sheepskin parchment; a unique biological resource that records the day, month and year of use (AD 1499 to 1969). We find these provide a high temporal resolution analysis of changing agricultural practices and episodes of disease. Most significantly, they suggest that if an ‘Agricultural Revolution’ occurred in livestock management, it did so from the mid-nineteenth century, in the aftermath of the Napoleonic Wars.

## Introduction

Historians agree the increase in British agricultural productivity between 1550 and 1880 was the result of major structural and technological innovations, notably: enclosure of open fields and commons, the adoption of new field rotation systems, the greater use of soil conditioners and fertilisers, and the improvement of livestock through selective breeding^1–4^. But this is where the consensus ends. Enduring disagreement as to when and how rapidly these developments occurred persists due to the lack of representative farming data, particularly for livestock^5–7^, prior to the start of annual agricultural returns in the 1860s. Consequently at least five periods of agricultural ‘revolution’ have been proposed^5^.

**Figure 1:**
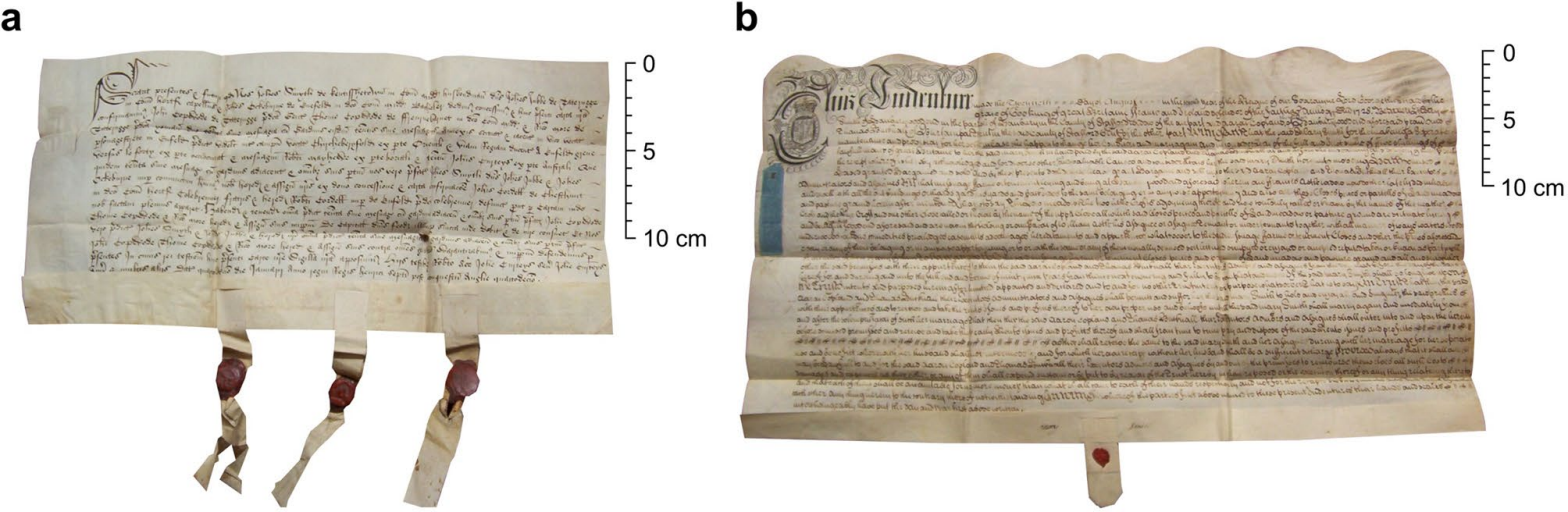
Legal deeds. (**a**) Title deed concerning the ownership of land in Enfield, Middlesex, signed and sealed 15th January 1499 (sample DL035); (**b**) title deed concerning property in Hanbury Woodend, Staffordshire, signed and sealed 11th August 1728 (sample DL110).

While the analysis of animal bones has provided valuable insights into the pastoral economy^8–10^, early-modern zooarchaeological assemblages are rarely of a chronological resolution that permits examination on a scale below that of a century. In contrast, historic legal documents written on sheepskin parchment (Fig. 1) record the day, month and year the agreement was signed; a date likely only a few months after the death of the animal from which the parchment was produced^11^. These documents provide an exceptional resource for high-resolution investigations of animal and land management strategies through stable isotope analysis, a tool used for reconstructing diet^12^, discriminating the use of organic and inorganic fertilisers^13^, exploring stocking densities^14^ and identifying transhumance^15^. Recent analysis demonstrates that parchment is a viable analyte for isotope analysis, recording dietary and physiological signals from the weeks and months before death^16,17^. Therefore to provide new insight on the timing, extent and drivers of agricultural change, we undertook the isotopic analysis (δ^13^C and δ^15^N) of 658 historic legal documents written on sheepskin parchment (Table 1).

**Table 1:** Collection information of parchment documents analysed in the study (*n* = number of samples). Collagen quality criteria outlined in the methods section. The artificial collections contain documents of different provenance, while the others have grown organically around a single property.

Collection	*n*		Date range (AD)	Collection information
	Total	Acceptable collagen quality		
Cheshire Records Office	15	15	1786–1813	Artificial collection of deeds concerning property in Cheshire
Campana et al.^54^	13	9	1730–1830	Artificial collection of various legal documents
Doherty Collection	8	8	1913–1940	Artificial collection of deeds concerning property across England and Wales
Hull History Centre	38	33	1596–1969	Artificial collection of deeds concerning property in the East Riding of Yorkshire
Lee Collection	254	245	1499–1907	Artificial collection of deeds concerning property in England, Wales and Scotland
Lincoln Records Office	9	9	1742–1907	Artificial collection of deeds concerning property in Lincolnshire
Lord Collection	50	48	1582–1893	Title deeds concerning Lower Winskill Farm, Settle, North Yorkshire
Tye Collection	254	253	1650–1904	Artificial collection of deeds concerning property in the City of London. Documents were discarded from the Sun Fire Office, London, company archives
Westminster City Archives	1	1	1707	Title deed from the City of Westminster
Wills Collection	16	14	1652–1790	Title deeds concerning property in Somerset
Total	658	635		

## Results and discussion

**Figure 2:**
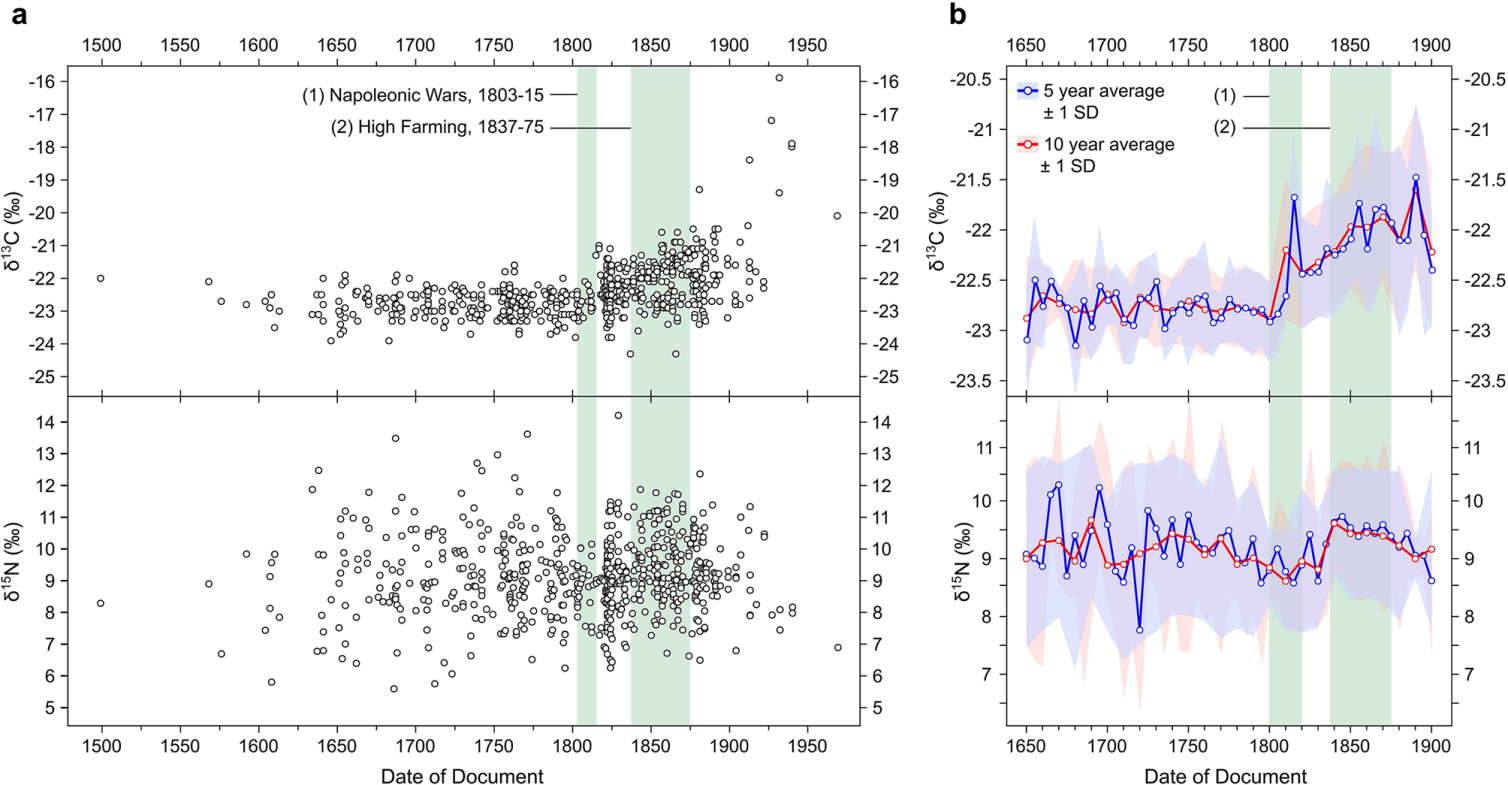
Isotope values from historic sheepskin parchment. (**a**) δ^13^C (top) and δ^15^N (bottom) of individual skins plotted against the year the document was signed; (**b**) 5 and 10-year averages of δ^13^C (top) and δ^15^N (bottom) from AD 1650 to AD 1900.

Full δ^13^C, δ^15^N and elemental composition results are presented in Supplementary Dataset 1, with summary statistics provided in Table 2, and plotted chronologically in Fig. 2. Of the 658 samples analysed, 23 failed to meet collagen quality criteria and were excluded from the analysis.

**Table 2:** Summary of δ^13^C and δ^15^N values by 25-year periods (*n* = number of samples). Due to the small sample sizes, parchment dated between AD 1499–1599 and 1925–1969 are presented as single groups.

Period	*n*	δ^13^C			δ^15^N		
		Mean (‰)	Range (‰)	SD	Mean (‰)	Range (‰)	SD
1499–1599	4	− 22.4	0.8	0.4	8.4	3.1	1.3
1600–1624	7	− 22.8	1.0	0.4	8.3	4.0	1.4
1625–1649	9	− 22.9	1.9	0.5	9.0	5.7	2.1
1650–1674	22	− 22.8	1.9	0.5	9.3	5.4	1.5
1675–1699	27	− 22.8	2.1	0.4	9.2	7.9	1.6
1700–1724	28	− 22.8	1.3	0.3	8.8	4.9	1.4
1725–1749	37	− 22.8	1.5	0.4	9.4	6.1	1.4
1750–1774	65	− 22.8	2.1	0.4	9.2	7.1	1.3
1775–1799	47	− 22.8	1.7	0.4	9.0	5.5	1.2
1800–1824	109	− 22.5	2.7	0.6	8.8	5.2	1.0
1825–1849	86	− 22.3	3.0	0.5	9.3	7.8	1.2
1850–1874	102	− 21.9	3.7	0.7	9.5	5.1	1.2
1875–1899	71	− 21.9	4.1	0.8	9.3	5.9	1.0
1900–1924	15	− 21.7	4.5	1.2	9.3	4.5	1.3
1925–1969	6	− 18.1	4.2	1.5	7.8	0.7	0.3

### Procurement of skins

Parchment produced δ^13^C values from − 24.3‰ to − 15.9‰ (mean: − 22.4‰). Except for seven skins from the late 19th (*n* = 1) and twentieth century (*n* = 6) with values higher than − 20‰, all are consistent with sheep raised in the British Isles grazing on C_3_ grasses in agreement with the documents’ provenance^18^. Those with values above − 20‰ indicate the consumption of C_4_ plants^19^, although with the global trade in both livestock and fodder in these later decades, it is difficult to say if it is the animal or their feed which are of a non-domestic origin.

Parchment produced δ^15^N values from 5.6‰ to 14.2‰ (mean: 9.1‰), with many higher than those typically observed in terrestrial herbivores. Sheep bone collagen from the same period has yielded δ^15^N values from 3.6 to 10.2‰ (mean: 6‰)^20,21^, therefore the elevated values in parchment can be attributed in part to inter-tissue isotopic discrimination (mean δ^15^N_(skin-bone)_ + 1.1‰^17^) and the impact of parchment production (mean δ^15^N_(skin-parchment)_ + 0.3‰^16^). Additionally, these values likely reflect the finishing of sheep on well manured crops and pasture in an effort to increase their weight before slaughter. Across the English downlands where the parchment making industry was principally located^11^, crop cultivation was supported by large flocks that were folded on fallow arable fields to enrich the thin soil with their dung^22^. To provide a concentrated covering, sheep were kept in moveable pens in high densities^23,24^; a practice with the potential to substantially elevate δ^15^N values in the crops and the consumer tissues^25,26^. This supplementary feeding over the last 6–8 weeks^27^ would not be detectable isotopically in bone collagen but would be in skin collagen due to its faster turnover rate^28,29^. More detailed assessment of regional patterns is not possible as the location the legal agreement concerns, or the stationer through whom the parchment was sold, typically bear no relation to the location the animal was raised^11^.

It is unlikely these high values reflect the use of young lambskins exhibiting a trophic enrichment from suckling, which elevates δ^15^N in the offspring’s tissues around 2‰ above that of the mother^30^. Lambs were typically weaned around 16–18 weeks old during this period^31^; too young to yield a skin of the ~ 70 × 50 cm typical of legal deeds. Legislation prohibiting the importation of leather gloves ensured lambskins were reserved for these and other expensive goods, while cheaper adult skins were used for parchment—as specified in contemporary manufacturing guides^32–34^.

### Episodes of famine and disease

The unusually high δ^15^N of some skins may indicate they come from sheep which had been in poor health before death. Acute physiological stress due to starvation or illness typically results in an elevation of δ^15^N due to catabolism of the body’s own protein resulting in a fractionation characteristic of trophic level increases^35,36^. While most nutritional stress studies have focussed on inert tissues, recent analysis demonstrates that the rapid turnover of skin collagen enables the recording of short-term stresses isotopically^17^.

A series of elevated nitrogen values coincide with an outbreak of ‘sheep-rot’ during the nineteenth century. Sheep-rot (bacterial pododermatitis) is a highly contagious and painful condition characterised by necrosis of the interdigital skin causing severe lameness, a reluctance to graze, emaciation, and ultimately death if untreated^37^. Epidemics appeared throughout the early-modern period, but a particularly virulent form spread across Britain between 1828 and 1831, killing an estimated 8 million sheep; a quarter of the national flock^38^. The twenty-one deeds covering these years have a mean δ^15^N of 9.6‰, comparable with that of all samples, but five of these (five separate documents across three collections) have δ^15^N values > 10.5‰, including one with an extraordinary high δ^15^N value of 14.2‰, the highest observed in all samples.

It is tempting to interpret these five as victims of the rot which experienced acute inappetence and physiological stress prior to death. The disease can take months to reach an advanced stage and persist in chronic form for a duration more than sufficient to be recorded isotopically in skin^39^. Contemporary accounts indicate sheep suffering from rot were sent to market as farmers sought to recoup their losses^40^, providing the opportunity for their skins to be sent to a parchment maker. Yet the skins showed no visible sign of malnutrition, such as an impression of the ribs due to excessive thinness^34^. Dearth and disease were perennial challenges for farmers prior to the advent of pesticides and antibiotics and one must be careful not to simply find historical accounts that support these anomalies. However this and other correlations invite future investigations of parchment for paleopathology studies using additional biomarkers of stress and nutritional deficiency.

### Agricultural change after the Napoleonic Wars

δ^13^C values between the early sixteenth century and early nineteenth century display remarkable uniformity, with almost all falling between − 24 and − 22‰. When grouped into 25-year periods, no statistically significant difference was observed between quarter centuries (Table 3), with just 0.1‰ variation in mean values between 1600–1624 and 1800–1824 (Table 2). In contrast, δ^15^N values exhibit a high degree of variability, although again with no statistically significant variation in mean values between 25-year periods.

**Table 3:** Significance of differences between 25-year periods in isotope values (Mann–Whitney *U* test). Number of samples in parentheses. *p < 0.05, **p < 0.01, ***p < 0.001.

Period	δ^13^C		δ^15^N	
	U	p	U	p
1650–1674 (22) vs. 1675–1699 (27)	273.5	0.64	265.5	0.53
1675–1699 (27) vs. 1700–1724 (28)	342.5	0.55	346.0	0.59
1700–1724 (28) vs. 1725–1749 (37)	495.0	0.76	422.5	0.21
1725–1749 (37) vs. 1750–1774 (65)	1153.0	0.73	1109.0	0.52
1750–1774 (37) vs. 1775–1799 (47)	1437.0	0.59	1446.5	0.63
1775–1799 (47) vs. 1800–1824 (109)	1815.5	0.004**	2334.5	0.38
1800–1824 (109) vs. 1825–1849 (86)	3695.0	0.01**	3669.0	0.009**
1825–1849 (86) vs. 1850–1874 (102)	2920.0	< 0.001***	3875.5	0.17
1850–1874 (102) vs. 1875–1899 (71)	3463.0	0.63	3213.5	0.21
1875–1899 (71) vs. 1900–1924 (15)	519.0	0.88	504.0	0.75
1900–1924 (15) vs. 1925–1969 (6)	2.0	< 0.001***	13.0	0.01**

The absence of statistically significant variation across the sixteenth to eighteenth century is surprising considering contemporary agricultural innovations, such as new field rotation systems which replaced fallows with grass leys and nitrogen-fixing root vegetables and legumes^5^. Biometrical data indicates an increase in the size of sheep across these centuries^8^, and as the dimensions of the appendicular skeleton are heavily influenced by diet^41^ it has been suggested that this size increase may reflect nutritional improvement^42^. Our results, as well as those of Fisher and Thomas^43^ for cattle, suggest that early size increases were not accompanied by isotopically detectable changes in environment or diet, and were more likely to have been the result of genetic modifications through the introduction of new stock and selective breeding.

A significant change in isotopic values occurred from the early-nineteenth century, with mean values for both δ^13^C and δ^15^N increasing across the nineteenth century (Table 3). There is considerable ambiguity in interpreting complex data of this nature, with multiple possible readings of this increase. We suggest this reflects the shift in animal and land management in the wake of the Napoleonic Wars (1803–1815). The war created boom conditions for arable farmers, stimulating an extension of the land under cultivation beyond even the limits of the Second World War’s reclamation campaign^44^. As hostilities ceased and the continental blockade lifted, the era of high prices was replaced by decades of deflation. With the fresh supply of cultivable land almost exhausted and innovations introduced during the seventeenth and eighteenth centuries now running into diminishing returns^7^, farmers sought new ways to enhance their profits and productivity. Many met the fall in prices with intensive mixed farming, known as ‘high farming’ or ‘high feeding’, which achieved high outputs by maintaining large numbers of livestock on imported feeds, producing more manure, which in turn increased soil fertility and ultimately grain yields.

Previous improvements had essentially involved the recycling of materials produced on the farm itself. The essence of what F.M.L Thompson termed the ‘Second Agricultural Revolution’ (1815–1880) was that “it broke the closed-circuit system and made the operations of the farmer much more like those of the factory owner”^4^, relying on inputs imported from outside the farm and indeed the country, particularly oil-cake fodder and bonemeal fertiliser^44,45^. Oil-cakes were a by-product of rape, linseed and cottonseed oil extraction. Virtually all were derived from imported materials, at first Prussia, then Russia from the 1820s, India from the 1850s, and Egypt from the 1860s^4^. American maize was added to oil cakes from the 1840s^45^. Their consumption increased substantially during the nineteenth century, growing from 35,000 tons in the 1820s to 740,000 in the 1880s. Crops grown in continental Europe are characterised by higher δ^13^C values than those grown in the British Isles, a difference which is recorded in the isotopic composition of the animal tissues^18,46,47^. The consumption of oil-cakes made from imported crops, including C_4_ maize, is likely the key driver in the increasingly higher δ^13^C values seen from 1815 onwards.

The growing use of organic fertilisers such as bonemeal in the nineteenth century is reflected in the significant elevation in mean δ^15^N between 1800–1824 and 1825–1849 (Table 3). Bones were either crushed into half-inch pieces, ground into bonemeal or dissolved in sulphuric acid to be converted into superphosphate^7^. The quantity of domestic and imported bones is estimated to have increased from 30,000 tonnes in the 1810s to 115,000 by the 1880s^4^. Even the bones of those who fell at Waterloo were ground for fertiliser, performing, as one farmer regarded, “the less glorious, but more useful purpose of producing wheat for their brothers at home”^48^. By the mid-eighteenth century, bones were supplemented with the nitrogenous Peruvian seabird guano and Chilean nitrates^7^ as increasingly distant sources of nutrients were used to replenish exhausted British fields. Despite the elevated δ^15^N values of marine bird guano (> + 26‰^49^) there is no clear evidence of the 1840–1880s ‘guano craze’ in sheepskins from this period, though contemporary surveys suggest it was infrequently used on the chalk downlands where the parchment making industry was principally located^50^.

## Conclusions

The isotopic compositions of sheepskin parchment indicate a substantial change in animal and land management from the second quarter of the nineteenth century. This transformation was driven by renewed and unfettered access to continental and American markets for manures and animal feed, which developed into ‘High Farming’ as agricultural production adapted to more intensive patterns of feeding and manuring. This quantitative data is a new and significant addition to the theory that there were staged episodes within a long ‘agricultural revolution’ much of which was contemporaneously with full industrialisation^51^. More broadly, the findings demonstrate that parchment is an extraordinary high-resolution biomolecular archive through which centuries of environmental history, agricultural history and animal health can be explored.

## Materials and methods

### Sampling

Samples were obtained from 645 individual skins from a total of 477 deeds. Of the deeds with multiple pages, each was of a size (> 70 × 50 cm) to indicate they came from a single animal. Samples (approx. 1 × 5 mm) were removed from the edge of each skin from an area devoid of any ink, stamp, glue, seal or surface marking to avoid contamination.

Species identification via peptide mass fingerprinting, presented in Doherty et al.^52^, identified 622 (96.4%) as sheep (*Ovis aries*), whilst the remaining 23 (3.6%) could be classified as sheep or goat (*Capra aegagrus hircus*) as separation between the species was not possible due to a lack of diagnostic peptides. Sheepskin was preferentially used over goat of calfskin (*Bos taurus*) for the production of legal documents from the twelfth century onwards in England, Wales and Ireland due to its susceptibility to delaminate when scrapped serving to deter fraudulent textual erasure and modification^52^. No British legal deed has been identified as goatskin through biomolecular analysis^52,53^. It is therefore highly likely that these 23 skins are from sheep and as such were included in the analysis.

Isotopic data from thirteen eighteenth and nineteenth century British legal deeds reported by Campana et al.^54^ have been included in all statistical analyses, bringing the total to 658. While the original DNA analysis could not determine the species, subsequent identification via peptide mass fingerprinting has identified these as sheep (pers. comm. M.J Collins). Samples were prepared following the same methodology as this study.

### Stable isotope analysis

Samples were prepared for stable isotope analysis at BioArCh facilities, Department of Archaeology, University of York, following the methodology outlined in Doherty et al.^16^. Lipids were removed via solvent extraction, dichloromethane/methanol (2:1 v/v), by ultrasonication for 1 h, with the supernatant removed and solvent replaced every 15 min. The samples were subsequently demineralised in 0.6 M HCI at 4 °C for 6 h to remove residual calcium carbonate/hydroxide, rinsed with distilled water, and gelatinised in 0.001 M pH 3 HCI at 80 °C for 48 h. The supernatant containing the collagen was filtered (60 μm Ezee-Filter™, Elkay Laboratories, UK), frozen and freeze-dried.

Prepared collagen (0.9–1.1 mg) was weighed out in duplicate in 5 × 3.5 mm tin capsules (Elemental Microanalysis, Okehampton, UK) and analysed at the Natural Environment Research Council Life Sciences Mass Spectrometry Facility (NERC LSMSF) in East Kilbride, UK, where isotope ratio determinations were carried out on a ThermoElectron DeltaPlusXP (Thermo Fisher Scientific, Bremen, DE) with an Elementar Pyrocube elemental analyser (Elementar UK Ltd). Sample data were reported in standard delta per mil notation (δ ‰) relative to V-PDB (δ^13^C) and AIR (δ^15^N) international standards. Three laboratory reference materials were interspersed within the measurement run to correct for linearity and instrument drift. Each of the laboratory reference materials is checked regularly against international standards USGS40 and USGS41. Following the calculations outlined in Szpak et al.^55^, the total analytical uncertainty was estimated to be ± 0.18‰ for δ^13^C and ± 0.20‰ for δ^15^N.

To account for the changing ratio of atmospheric ^13^CO_2_ to ^12^CO_2_ due to increased anthropogenic fossil fuel emissions, parchment δ^13^C values were corrected following Dombrosky’s^56^ Suess model to the average atmospheric δ^13^C of AD 1760 (− 6.4‰), prior to the Industrial Revolution (Supplementary Dataset 1). This atmospheric correction increased the amount of ^13^C in samples after AD 1807 by 0.1–0.9‰.

### Collagen quality indicators

Parchment produced an average collagen yield of 69.4% (range 32.4–98.3%), and %C ranged from 37.1 to 47.3% and %N from 12.3 to 16.9%; all consistent with modern parchment and skin^16^. Samples produced C:N ratios ranging from 3.1 to 3.5. Skin collagen has a theoretical C:N ratio of 3.11^16^ and the elevated ratios in parchment are likely due to collagen hydrolysis during the liming process. Twenty three samples produced C:N ratios > 3.5 and were excluded from the analysis.

### Statistical analysis

Statistical testing was carried out using the IBM SPSS Statistics 27 software package. Shapiro-Wilks test for normality indicated δ^13^C data did not conform to a normal distribution (P < 0.05), thus the resultant statistical tests were non-parametric in nature. Significance of difference in isotope values between period 25-year period groupings were evaluated with a Mann–Whitney *U* test.

### Ethical compliance

No experiments were performed on live animals, or animal tissue deriving from previous experiments. The authors were not involved in the life or death of animals from which the parchment was made.

## Supplementary Information

Supplementary Information.

## Data Availability

All data generated in this study are presented in the article and Supplementary Information file.
